# The relationship between principals’ pedagogical leadership practices and teachers’ job performance: the role of sociodemographic characteristics

**DOI:** 10.1186/s40359-025-02415-7

**Published:** 2025-01-30

**Authors:** Kelemu Zelalem Berhanu

**Affiliations:** Department of Education Leadership and Management, Faculty of Education, University of Johannesburg, Johannesburg, South Africa

**Keywords:** Age, Education level, Gender, Job performance, Length of service, Pedagogical leadership

## Abstract

**Background:**

Concerns about leadership style and job achievement are global priorities. Most previous investigations have concentrated on one or two of the variables rather than on the sociodemographic characteristics. To properly understand the impact of leadership styles on teacher performance, it is critical to grasp the sociodemographic characteristicsvia a holistic approach to rethinking and improving the existing level.

**Objective:**

This study seeks to investigate the relationship between principals’ practice of pedagogical leadership style and teachers’ job performance by considering the effects of sociodemographic characteristics.

**Methods:**

A cross-sectional survey research methodology was used, and in Ethiopia’s Gozamin and Dembecha districts, 539 teachers participated in the study.

**Findings:**

Consequently, there were strong and moderate correlations between teachers’ job performance and pedagogical leadership. Sex did not significantly impact principals’ pedagogical leadership practices or teachers’ job performance. However, age and length of service significantly influenced principals’ pedagogical leadership practices but not teacher performance. Education level significantly affected teachers’ job performance but not leadership practices.

**Conclusions and implications:**

- principals’ practice of pedagogical leaders plays a predictive role in teachers’ job performance. Specific sociodemographic factors also have a role in shaping leadership perceptions and performance outcomes. This study added to the body of knowledge in the fields of education and leadership about work performance and leadership style. In the end, the study inspired stakeholders in schools, government decision-makers, and other researchers to continue improving teachers’ jobs’ effectiveness by identifying factors influencing their success. These results can potentially make teachers more accessible to more students and schools and thus contribute to ensuring quality education (SDG 4).

## Introduction

Human capital, or employee job performance, is the most crucial element in achieving institutional goals, even if workforce competency, financial capital, and technical capital are all crucial. Likewise, the total effectiveness of every school is dependent on the performance and empowerment of its instructors. Various scholars have characterized how well instructors do their duties. One definition of work performance given by Motowidlo and Kell [1] is the total expected value to the organization of the individual’s discrete behavioral episodes over a predetermined period. Here, “performance” solely refers to actions that might affect the accomplishment of corporate goals. The performance domain encompasses good and poor behaviors that impact the attainment of business objectives. Nelson and Quick [2] also described work performance as completing specific educational assignments. Subsequently, Tehseen and Hadi [3] defined teacher performance as the capacity of educators to provide outstanding outcomes, finish assignments on schedule, be present at work, and work with other educators. This definition of work performance is a slightly modified version of the one that was previously published in a study. The current study takes advantage of this as well. Teaching performance shows how successfully teachers manage and collaborate with students while fulfilling their professional and instructional obligations [2, 3].

Teacher job performance (JP) is affected by several factors. Students’ education and the accomplishment of school goals are impacted by poor teacher performance [3]. Teachers’ psychological empowerment (PE) determines their work performance (JP) [4–8]. The ways administrators lead have an impact on teachers’ work performance. Principals have a wide range of leadership philosophies in the school setting, depending on the school culture [9]. A school leadership model may use transformational, moral, and instructional leadership approaches to promote school performance and accomplishment [9]. Furthermore, Bose et al. [10] discovered a high association between favorable organizational results and leadership style. School administrators must thus know how to employ the leadership style that best fits the requirements of educational establishments.

It is necessary for pedagogical leaders to integrate several styles (such as personality, behavioral, and skill approaches) and a proper leadership strategy across different situations [11, 12]. In order to assist teachers in accomplishing school goals, successful school principals employ suitable leadership techniques or a mix of techniques [13]. One effective leadership style for accomplishing educational institutions’ goals in the twenty-first century is pedagogical leadership [14]. By assisting teachers and students in becoming inquisitive, compassionate, and focused communities, pedagogical leadership, as defined by Sergiovanni [14], is an investment in the growth of their social, intellectual, professional, and academic capitals. Leithwood et al. [15] stated that leadership in education is about influencing and coordinating people to accomplish school objectives. Regarding the relationship between JP and pedagogical leadership, Tirado-Calderón et al. [16] found that pedagogical leadership is an ongoing process of enhancing teachers’ skills and well-being. Although pedagogical leadership is essential to teaching, there is little research on how it affects teacher outcome behaviors directly and indirectly [16].

Recent studies, like those of Zaid et al. [17], emphasize the interaction effects between demographic factors like age, sex and total length of service on teachers’ job performance and perceived principals’ leadership practice. Teachers’ work effectiveness is also influenced by sociodemographic factors such as age, sex, length of service, and educational level. For example, Boon and Ganesan [18] found that attitudes toward leadership styles are broadly similar between the sexes in educational contexts. Because of their broader professional experience, older educators may value collaborative leadership more, according to Aina and Adebayo’s [19] and Berhanu’s [20] research. These findings suggest that a reexamination of the relationship between the style of pedagogical leadership and the performance of teachers in Ethiopia by taking sociodemographic characteristics into consideration is necessary to confirm or refute the empirical findings that have already been made available by several scholars. Furthermore, this study aims to fill this information gap about the factors influencing Ethiopian teachers’ working conditions. This significant research study offers valuable insights into the connections between pedagogical leadership styles and the JP of Ethiopian teachers.

District education office chiefs, school administrators, and other stakeholders in the educational institutions under investigation can benefit from this research’s insights into teachers’ work performance and the pedagogical leadership style of principals. Their awareness of the specific leadership style linked to teachers’ job performance will be beneficial. The plans of the educational institution will be rearranged to enhance teachers’ performance, which will promote students’ academic success, based on the study’s practical consequences and suggested future directions. The study’s conclusions pave the way for further research, especially in the area of the importance of including physical education while analyzing the performance of teachers in Ethiopian environments in connection to their pedagogical leadership style.

## Review of the literature

### The effects of sociodemographic variables and interactions on pedagogical leadership practices and teachers’ job performance

Sociodemographic characteristics including sex, age, duration of service, and educational attainment also have an impact on teachers’ job performance. For instance, Boon and Ganesan [18] discovered that, in educational settings, opinions about leadership styles are mostly comparable between the sexes. Similarly, current research supports the nonsignificant impact of sex on teachers’ work performance and pedagogical leadership methods. According to research by Liu et al. [21], professional development and job expectations frequently eclipse gender differences, confirming that gender does not necessarily impact leadership effectiveness or teaching results.

According to Aina and Adebayo’s [19] research, older educators may place a higher importance on collaborative leadership because of their broader professional experience. However, vitality, flexibility, and institutional support are frequently more important factors in work performance indicators than age alone [22]. According to Kaden et al. [23], seasoned educators anticipate independence and teamwork, consistent with successful pedagogical leadership. The lack of impact on work performance, however, is in line with a study by Jamil and Hamid [24], who discovered that motivation and ongoing professional growth are more important than experience alone in ensuring improved performance.

Regarding the impacts of educational attainment, previous scholars revealed that while it does not directly affect how instructors see leadership practices, higher educational attainment enhances teaching strategies and classroom results, which enhance job performance [25]. Recent research, such as Zaid et al. [17], highlights the infrequent independent behavior of demographic characteristics like sex and age, and the interaction effects between these variables and overall duration of service. Rather, their combined influence shapes professional practices and attitudes toward leadership. This interaction highlights the necessity of sophisticated leadership techniques catered to various teacher profiles.

The Ethiopian Ministry of Education provides several initiatives to empower educators, such as creating an attractive career development framework, designing in-service professional development training, and providing teachers [26]. Through various initiatives and motivations, the Ethiopian government hopes to assist educators in fostering their students’ success [27]. According to research from the Ethiopian education development plan (2018–30), poor teacher motivation and empowerment, significant teacher turnover, and insufficient school leadership in certain schools remain the main obstacles to teaching, even after several interventions [28]. Experienced teachers tend to adapt better to diverse leadership styles and contribute more effectively to educational outcomes, while younger teachers often require additional mentoring [29]. It is advised that research be carried out to examine how sociodemographic characteristics of teachers might predict their work performance in the context of Ethiopia, given the gaps and areas of dispute described above. Thus, hypothesis 1 was put up by the researcher:

#### H1

*Teachers’ sociodemographic characteristicsaffect teachers’ perceived pedagogical leadership practice of principals and their job performance.*

### The relationship between leadership style and teacher job performance

According to Dewettinck et al. [30], how leadership styles affect job performance is unclear. To enhance teacher performance, school leadership can set directions, offer counseling services, mentoring and coaching to improve advancing teacher performance, and support teachers in addressing organizational difficulties [9, 31]. Okoji [32] actively advocated merging authoritarian and democratic leadership approaches to enhance teachers’ JP. In some circumstances, it might be beneficial to combine authoritarian and democratic leadership styles. Combining authoritarian and democratic forms of leadership can be advantageous in certain situations. Autocratic leadership philosophies are distinguished by individual authority over all decisions and minimal involvement from group members. It is often most successful in situations requiring rapid decision-making. Democratic leadership philosophies, on the other hand, involve delegating decision-making tasks to members of the organization while fostering freedom of expression and collaboration. The concept of combining various approaches implies that a leader can adjust to the needs of the scenario [32]. Although authoritarian leadership had a negative and negligible impact on performance, Imhangbe et al. [33] discovered that democratic and laissez-faire leadership had a positive effect. Laissez-faire leadership is characterized by the leader’s limited engagement in decision-making processes. In schools, this technique may lead to principals being more of an observer, while teachers can do this autonomously, resulting in strong work ownership and performance [33]. Parveen et al. [34] stated that the variations in previous findings provide empirical evidence that primary leadership’s effects on JP depended on context. Task performance [35, 36] and teacher burnout [37] are two of the many mediation variables that are highlighted in the indirect relationship between leadership styles and work performance. These empirical studies focused on transformational, inclusive, ethical, transactional, and/or servant leadership styles. None of them placed any focus on pedagogical leadership. To fill this gap in the research and prior findings, the researcher established hypothesis 2 as follows:

#### H2

*Pedagogical leadership style is positively correlated with teachers’ JP*.

### Theoretical framework

Several models and theories justify the relationships between the variables [38, 39, 40]. This study followed Herzberg et al.‘s two-factor theory [39]. Herzberg’s Two-Factor Theory separates the variables affecting employee performance and motivation into two groups: motivators and hygiene elements. External variables that do not directly inspire employees, such as pay, working conditions, and the manager’s leadership style, can still lead to discontent if they are not met. Conversely, internal elements known as motivators are what propel workers to greater levels of happiness and performance. This study incorporates Herzberg’s paradigm by investigating the effects of extrinsic variables, such as a manager’s leadership style, and internal elements, such as sociodemographic traits, such as age, gender, education, and experience, on employee performance. It implies that although a leader’s style influences the workplace and keep people happy, internal elements like a worker’s motivation for success or development are essential for encouraging engagement and improving performance. This study integrates Herzberg’s framework by examining how intrinsic factors, such as sociodemographic characteristics (e.g., age, gender, education, and experience), and extrinsic factors, like a manager’s leadership style, impact employee performance. It suggests that while leadership style shapes the work environment and prevents dissatisfaction, intrinsic factors, such as an employee’s drive for achievement or growth, are critical for fostering engagement and improving performance. This theoretical consideration suggests that leadership style of leaders is one facet of a teacher’s work performance. According to these views, better performance can only be achieved when leaders and employees have reciprocal behavioral influences, a fit, and reasonable expectations [41]. This theoretical framework explains how personal and contextual variables interplay to influence teachers’ performance and adds unique insights to organizational and leadership theory [42].

## Methods

In order to gather data on the present problem and attempt to link factors that they were unable to control, the researcher employed the cross-sectional survey design. With this strategy, the researcher looks at the relationship between variables without making any changes by collecting data at a time [43]. The cross-sectional survey design makes it possible to identify the existence and degree of association between JP, sociodemographic information, and pedagogical leadership style.

### Participants

The Gozamen and Dembecha districts (woredas), located in the eastern and western Gojjam administrative provinces, respectively, were the study’s locations. Purposive sampling was used in the sample selection process to pick these two districts since they are exemplary in their respective provinces. It enables the researcher to obtain more accurate study findings and deeper insights. The results are pertinent to the research setting since the researcher only gathers data from the most appropriate people. Purposive sampling, according to Babbie [44], enables the researcher to choose the sample based on his own assessment and understanding of the population. Second, a thorough selection ensures that all 577 instructors across two districts are included in the research. Just 539 (93.4%) of them finished the survey.

### Instruments

Teachers were surveyed using two Likert scales, which are easy to construct and take minimal time to complete. The Likert scale ranges from 1 (strongly disagree) to 5 (strongly agree) for each measure.

**Teacher’s job performance** was adapted from Berhanu [45], who confirmed this in the Ethiopian setting. One of the seven things is “I took on challenging work tasks, when available.” This article evaluated construct validity using exploratory factor analysis (EFA). The item had the lowest factor load value (0.617), and the KMO was 89. The variance was 67.6% overall, which satisfies the requirements of EFA [46]. The researcher used a confirmation factor analysis (CFA) to examine the fitness of the job performance of the scale for the present data. The CFA results show that the fitting indices are within an acceptable range (see Table 1). Cronbach coefficient alpha was used to assess reliability. As a result, the alpha coefficient of the scale is 0.92, which indicates a high degree of reliability [47].

**Pedagogical Leadership**–was adapted from existing research [20]. The scale has 32 items. (e.g., “My principal creates a caring and supportive school culture.” According to EFA, the scale values’ total variance explained index is more than 50%, meeting the requirements. The measure shows construct validity, according to the confirmatory factor analysis. Chi-square (x2)/df = 3.19 is a basic way to check the goodness of fit index. The scale has a moderate degree of goodness of fit since x2/df is less than five. IFI, RFI, NNFI, and CFI indices greater than 0.95 indicated a favorable match. Overall, Cronbach Alpha was 0.89. The PL scale is, therefore, quite reliable [47].

The fit indices of the whole scales are offered precisely in Table 1 as per Schermelleh-Engel et al’s [48] goodness of fit measures.

**Table 1:** CFA results

Criteria	Acceptable Boundary /Criteria	Fitness of the model		
		pedagogical leadership		JP
X2/SD	≤ 2 = Perfect	X2/SD = 3.19	X2/SD = 2.5	
	≤ 2,5 = Perfect (Small)			
	≤ 3 = Perfect fit(large population)			
	≤ 5 = Moderate fit			
RFI/ NFI/ GFI/ CFI/ AGFI/ NNFI/IFI	≥ 0.95 = Perfect	NNFI = 0.98, RFI = 0.97, NFI = 0.97, IFI = 0.98, GFI = 0.88, AGFI = 0.85,CFI = 0.98	IFI = 0.92,NNFI = 0.96,GFI = 0.87,AGFI = 0.91,NFI = 0.94, RFI = 0.96, CFI = 0.98,	
	≥ 0.90 = Good fit			
RMSEA/RMR/SRMR	≤ 0.05 = Perfect	RMSEA = 0.071, RMR = 0.058 SRMR = 0.043	RMSEA = 084 RMR = 0.035 SRMR = 0.049	
	≤ 0.08 = Good			
	≤ 0.10 = Weak fit			

### Data collection procedures and data analysis

Several steps were taken in this study in order to validate the hypotheses and produce accurate and dependable results. Following obtaining the institutional review committee’s ethical approval, the researcher first piloted instruments with authorization from pilot schools and instructors. After the respondents gave their informed consent, the researcher began gathering pilot data after outlining the study’s purpose. The final scales were then distributed to the teachers. The teachers gave their own agreement responses for each topic. Respondents were receiving extensive assistance from data collectors to address any ambiguities. In this research, two data collectors were selected by the researcher because they were familiar with the culture, language, and norms of the two selected districts. Data collectors were teachers who taught in secondary schools in the Dembecha and Gozamen districts. Teachers from secondary schools in the Dembecha and Gozamen areas served as the data collectors. Lastly, SPSS-25 software was used for data analysis. Using the SPSS 25 software, Spearman correlation and descriptive statistics (such as frequency, percentage, and averages) and MANOVA and regression were employed. To confirm the structural validity and fitness of the scales model (CFA), Lisrel 7.4 software was utilized.

### Findings

The personal characteristics of the teachers are summarized in Table 2.

**Table 2:** Personal characteristics of the sample

Demographic information	groups		*N*	%
Gender		Male	424	78.7
		Female	115	21.3
Academic educational status		First degree	436	80.9
		Second degree	103	19.1
Teaching experience		< 10	121	22.4
		10–20	347	64.4
		> 20	71	13.2
		Total	539	100.0

Table 2 demonstrated that out of 577 individuals, 539 (93.4%) gave their complete data and took part in the research on a voluntarily basis. The remaining surveys, meanwhile, were incomplete. Regarding education level, the great majority of those surveyed held BA degrees. Ethiopia’s new education sector strategy [28] states that most teacher certifications are insufficient since secondary institutions demand a master’s degree.

### Relationship between pedagogical leadership and teachers’ job performance

The views of the teachers on the relationship between principals’ practice of pedagogical leadership style and teachers’ job performance are presented in Table 3.

**Table 3:** The relationship between pedagogical leadership and JP

	Mean	1	2
1. Pedagogical Leadership Practice	3.7640	1	
2. Teachers’ Job Performance	3.6878	0.396**	1

To determine status of principals’ practice of pedagogical leadership and teachers’ job performance, the following criteria were applied: the means of greater than 3.67 is high; between 2.34 and 3.66 is moderate; below 2.34 is low [49]. As a result, teachers have high job performance and a high perception towards principals’ practice of pedagogical leadership style. Additionally, Table 3 showed a somewhat moderate and positive relationship (*r* = 0.396, *p* < 0.01) between principals’ pedagogical leadership practices and teachers’ job performance. The analysis of Cohen’s [50] correlation interpretation placed the study’s variable relationships in the moderate to strong range (small, < 0.30; medium, between 0.3 and 0.5; large, > 0.5). It is possible to state that a rise in one research variable will result in an increase in other variables in this way.

**Table 4:** The effect of sex, age, total length of service and education level on linear association between pedagogical leadership practice and teachers’ job performance

Sociodemographic characteristics or independent variable	Dependent Variable	Type III Sum of Squares	df	Mean Square	F	Sig.	Partial Eta Squared
Sex	Pedagogical leadership	0.772	1	0.77	1.82	0.178	0.004
	teacher performance	0.819	1	0.81	0.95	0.329	0.002
Age	Pedagogical leadership	7.046	3	2.34	5.54	0.001	0.032
	teacher performance	3.629	3	1.21	1.40	0.23	0.008
Total service of teaching	Pedagogical leadership	5.468	2	2.73	6.45	0.002	0.025
	teacher performance	0.191	2	0.09	0.11	0.89	0.000
Level of education	Pedagogical leadership	1.251	1	1.251	2.95	0.08	0.006
	teacher performance	10.424	1	10.42	12.14	0.001	0.023
Sex * Age	Pedagogical leadership	6.448	3	2.149	5.07	0.002	0.029
	teacher performance	3.728	3	1.243	1.44	0.22	0.008
Sex * Total service of teaching	Pedagogical leadership	6.292	2	3.146	7.42	0.001	0.028
	teacher performance	4.364	2	2.182	2.54	0.08	0.010
Sex * Level of education	Pedagogical leadership	1.286	1	1.286	3.036	0.082	0.006
	teacher performance	1.066	1	1.066	1.242	0.266	0.002
Age * Total service of teaching	Pedagogical leadership	11.068	6	1.845	4.355	0.000	0.049
	teacher performance	15.060	6	2.510	2.924	0.008	0.033
Age * Level of education	Pedagogical leadership	6.296	3	2.099	4.954	0.002	0.028
	teacher performance	6.053	3	2.018	2.351	0.072	0.014
Total service of teaching * Level of education	Pedagogical leadership	1.241	2	0.621	1.465	0.232	0.006
	teacher performance	2.649	2	1.324	1.543	0.215	0.006
Sex * Age * Total service of teaching	Pedagogical leadership	0.040	1	0.040	0.093	0.760	0.000
	teacher performance	0.403	1	0.403	0.469	0.494	0.001
Sex * Age * Level of education	Pedagogical leadership	0.000	0	.	.	.	0.000
	teacher performance	0.000	0	.	.	.	0.000
Sex * Total service of teaching * Level of education	Pedagogical leadership	0.374	1	0.374	0.882	0.348	0.002
	teacher performance	0.205	1	0.205	0.239	0.625	0.000
Age * Total service of teaching * Level of education	Pedagogical leadership	0.767	2	0.384	0.905	0.405	0.004
	teacher performance	1.089	2	0.545	0.635	0.531	0.002
Sex * Age * Total service of teaching * Level of education	Pedagogical leadership	0.000	0	.	.	.	0.000
	teacher performance	0.000	0	.	.	.	0.000
Error	Pedagogical leadership	215.608	509	0.424			
	teacher performance	436.950	509	0.858			
Total	Pedagogical leadership	7902.13	539				
	teacher performance	7857.06	539				
Corrected Total	Pedagogical leadership	265.622	538				
	teacher performance	526.543	538				

a. R Squared = 0.188 (Adjusted R Squared = 0.142)

b. R Squared = 0.170 (Adjusted R Squared = 0.123)

Table 4 indicated the effects of independent variables (sex, age, total length of service, and education level) and their interactions on two dependent variables: pedagogical leadership practice and teachers’ job performance using MANOVA. On main effects, Table 4 showed sex has no significant effect on either pedagogical leadership (F = 1.822,*p* = 0.178) or teacher performance (F = 0.954,*p* = 0.329). Age has significant effect on teachers’ perceived principals’ practice of pedagogical leadership (F = 5.545,*p* = 0.001, partial eta squared = 0.032), however, there was no significant effect on teacher performance (F = 1.409,*p* = 0.23). Total length of service has also significant effect on pedagogical leadership (F = 6.455, *p* = 0.002, partial eta squared = 0.025) while it has no significant effect on teacher performance (F = 0.111,*p* = 0.895). Education Level has no significant effect on pedagogical leadership (F = 2.954,*p* = 0.086) while it has significant effect on teacher performance (F = 12.143,*p* = 0.001, partial eta squared = 0.023).

Partial eta squared values indicated small to moderate effect sizes for significant factors. Age’s effect on pedagogical leadership (η2 = 0.032) is small. Education level’s effect on teacher performance (η2 = 0.023) is also small. To sum up, significant predictors for pedagogical leadership: Age, total service, and some two-way interactions (e.g., sex × age, age×total service). Significant predictors for teacher performance were education level and the interaction of age × total service. This table highlights the complexity of relationships between demographic variables and teacher outcomes, emphasizing the importance of targeted analysis of age and service-related factors in educational settings.

**Table 5:** The effects of principals’ pedagogical leadership practice on teachers’ job performance

Model			Coefficients			t	Sig.
		R^2^	B	Std. Error	Beta		
1	(Constant)		1.590	0.214		7.44	0.00
	Pedagogical leadership	0.157	0.557	0.056	0.396	9.98	0.00

N.B. Dependent Variable: teacher performance; ANOVA results; F (1, 537) = 99.732, *p* < 0.001

The regression Table 5 revealed that about 15.7% of the variance in teachers’ job performance is explained by principals’ pedagogical leadership practices. While this is a statistically significant result, it suggests other factors also contribute to teachers’ performance. The ANOVA also assesses whether the overall regression model is statistically significant: The model is highly significant, meaning pedagogical leadership practices significantly predict teachers’ job performance (F (1, 537) = 99.732, *p* < 0.001). The impact of principals’ pedagogical leadership approaches on teachers’ work performance is assessed in the regression table. Even though this conclusion is statistically significant, it implies that teachers’ job performance is influenced by other factors as well. Additionally, the ANOVA determines if the regression model as a whole is statistically significant. The model is very significant, indicating that teachers’ job performance is considerably predicted by pedagogical leadership methods (F (1,537) = 99.732, *p* < 0.001).

In general, based on Tables 4 and 5, hypotheses 1 and 2 were found to be supported.

## Discussion

This study investigated the role of pedagogical leadership in predicting teachers’ job performance by taking the social exchange theory, the two-factor theory, the job demands–resources model, and the social cognitive theory as theoretical frameworks. For instance, the JDR model [51] offers a thorough comprehension of how many antecedents interact with extrinsic demands, resources, and results. The JD-R model states that both internal and extrinsic factors—such as the principals’ pedagogical leadership style and sociodemographic characteristics—have an impact on the study’s result, or the job performance of the teachers. Teachers’ opinion was high for the composite scores of principals’ practice of pedagogical leadership, and job performance of teachers. While there are several reasons why the present study’s teachers have performed better, one factor could be the effective principals’ pedagogical leadership practices. In contrast to the current study, Afework [52] has shown that principals are not competent enough to use contemporary leadership theories and practices. According to sources, the Amhara National Regional State in Ethiopia’s secondary school performance falls short of expectations [53].

The present study revealed that some teachers’ demographic characteristics had an impact on their work performance and how they regarded the pedagogical leadership practices of their principals. Similar to the current study, Aina and Adebayo’s [19] findings on age showed that older educators would place a higher priority on collaborative leadership because of their wider exposure to the workplace. However, energy, flexibility, and institutional support are frequently more important factors in work performance indicators than age alone [22]. The notion that experienced teachers frequently choose participative leadership approaches is supported by their teaching experience with regard to pedagogical leadership techniques. However, the lack of influence on job performance is consistent with research by Jamil and Hamid [24], which found that experience alone does not guarantee higher performance; rather, motivation and continuous professional development are essential. The significant influence of education level on teachers’ job performance but not on leadership perceptions is consistent with recent research. Kim and Lee [25] demonstrated that higher educational attainment improves teaching methods and classroom outcomes but does not directly impact how teachers perceive leadership practices. This may be because leadership perception depends more on relational dynamics than formal qualifications.

However, gender had no significant effect on teachers’ job performance and teachers’ perceived principals’ practice of pedagogical leadership. In same the vein, Boon and Ganesan [18] found that perceptions of leadership styles are generally similar across genders in educational contexts. In same way, research by Liu et al. [21] confirms that gender does not inherently determine leadership effectiveness or teaching outcomes, as professional development and role expectations often overshadow gender differences. Therefore, hypothesis 1 was partially supported by current data.

The present study showed that pedagogical leadership and job performance of teachers had moderate and significant relationship. The regression result also revealed that principals’ practice of pedagogical leadership style predicted teachers’ job performance. In the same vein, studies have also revealed that the leadership style positively predicted the JP of teachers [31, 33, 45]. According to Bickmore and Dowell [54], an unfavorable leadership style could discourage teachers from achieving mutual goals. Parveen et al. [34] stated that the variations in previous findings provide empirical evidence that the effects of primary leadership on JP were dependent on context. According to Joyce et al. [55] and Parveen et al. [34] autocratic and democratic leadership styles have a favorable impact on JP. However, laissez-faire leadership style affected teachers’ job performance negatively.

On the other hand, Joyce et al. [55] found no noticeable impact of a laissez-faire leadership style on employee performance. Additionally, Luo et al. [40] found that teachers’ job performance is considerably and favorably influenced by perceived inclusive leadership. Okoji [32] also strongly recommended combining autocratic and democratic leadership styles to improve job performance. Similarly, Hallinger and Heck [53] demonstrated that pedagogical leadership fosters teacher collaboration, improving job performance. When school leaders focus on the pedagogical components of education, teachers’ performance improves, most likely because such leadership styles promote teachers’ professional development and align with their empowerment in student learning. When employees perceive leaders’ leadership practice as meaningful, it aligns with an individual’s values and motivation [56]. Robinson et al. [57] also found that the leadership style of school leaders significantly enhances teacher effectiveness, directly affecting performance. These research investigations focused on servant, transactional, ethical, inclusive, and/or transformative leadership approaches. According to the JDR model and social exchange theory, better performance can be achieved if there is a logical degree of expectation fit and positive influence between leaders and employees. Hence, the present study findings were also supported by JD-R model and social exchange theories. To summarize, the current study found that pedagogical leadership acted as a predictive factor for teacher job performance.

### Limitations and suggestions for future scholars

This study has limitations, first, a limited number of Ethiopian districts and a generalizability problem. Therefore, in order to generalize to a wide population, further research is required to examine the link between teachers’ performance under different conditions and their pedagogical leadership style across several districts. Second, in the present study, perceptual data, such as administrators’ pedagogical leadership style and teacher evaluations of their own work performance was included. Common source bias, however, may have happened. Future researchers can thus use a range of data gathering techniques to lessen individual bias. Third, the current cross-sectional survey study merely identifies relationships; it does not determine the cause and effect between variables and lacks control over them. This indicates that extraneous variables may interfere with the information. Therefore, future studies should take into account longer experimental designs to investigate causal correlations, as well as more behavioral data gathered from diverse sources. Fourth, the use of purpose sampling may add selection bias because the districts were chosen based on assumptions provided by the researcher. For example, the sample may not be representative of the larger community, and the findings acquired through purposive sampling may be limited in generalizability due to the unique selection criteria applied. As a result, future researchers can employ multistage or random sample approaches to simultaneously assess the effectiveness of the intervention of the study variables. Ultimately, among the most successful forms of leadership in educational institutions and systems is pedagogical leadership. Future scholars can carry out their studies to ascertain the impact of pedagogical leadership practice on schools’ objectives. In short, because of these limitations, the study’s findings on the correlations between study variables in this study cannot be generalized.

Despite these limitations, this study has some strengths that should be highlighted. First, this study expanded on previous research by investigating the relationship between pedagogical leadership, work performance and associated demographic characteristics. Second, in this investigation, the scales utilized to collect data were psychometrically validated. To summarize, this research underlines the importance of personal resources and leadership style in achieving improved workplace performance.This study is significant for school administrators, government, and other school stakeholders since it guides administrators and educators in supporting pedagogical leadership styles and improving teacher performance.

## Conclusion and implications

The results of the current investigation showed that teachers’ job performance was predicted by pedagogical leadership. As a result, education managers and policymakers can be proactive in encouraging school leaders to practice a pedagogical leadership style. This study makes theoretical and managerial contributions. This study’s original objective was to provide comprehensive knowledge of school leaders’ pedagogical leadership techniques and how they directly impact teachers’ job performance. The effort advances our understanding of education and leadership. Additionally, the study’s conclusions are applicable at the individual and organizational levels. The results have important management ramifications for comprehending and controlling factors that might lead to higher levels of performance at work on an individual basis. This suggests that if school administrators wish to enhance teachers’ job performance, they might select and implement pedagogical leadership styles. As a result, school administrators have the ability to establish an organizational culture that fosters productive relationships among coworkers as well as a cheerful, upbeat, and joyful work environment.

At the organizational level, first, the findings may be used as a basis for plans, policies, and initiatives that enhance job performance and pedagogical leadership practice. The results of this study suggest that policymakers should prioritize and support school leaders in developing their leadership skills, especially by including a variety of pedagogical leadership practice options into in-service training. Therefore, this study may prompt policy makers to change the way school principals work by advocating the use of pedagogical leadership styles. Second, it is anticipated that the study findings will be useful to school stakeholders, including administrators, parents, teachers, students, and legislators. Finding and correcting the many elements that affect teachers’ work performance might also help students perform better. Students’ performance can also be enhanced by identifying and addressing many factors that influence teachers’ job performance. Third, the results of this study will also help secondary schools and district education offices in Ethiopia’s Dembecha and Gozamen districts evaluate the productivity of their teachers. Additionally, the findings may have a more beneficial influence on continuing efforts to increase teachers’ work performance.

## Data Availability

No datasets were generated or analysed during the current study.

## Abbreviations

SDG 4: Sustainable Development Goal 4
JP: Job performance
